# Reactive Oxygen Species as Central Coordinators of Organelle Stress and Tumor Radiosensitivity

**DOI:** 10.1002/cbf.70324

**Published:** 2026-10-21

**Authors:** Guangyu Ju, Zimeng Wang, Jingjing Xiao, Qinxue Wu, Zhiying Yang, Yuqing Miao, Shuanghu Yuan, Junchao Qian

**Affiliations:** ^1^ Department of Radiation Oncology, The First Affiliated Hospital of USTC, Division of Life Sciences and Medicine University of Science and Technology of China Hefei Anhui P. R. China; ^2^ Anhui Province Key Laboratory of Medical Physics and Technology, Institute of Health and Medical Technology, Hefei Institutes of Physical Science, Chinese Academy of Sciences Hefei P. R. China; ^3^ Department of Radiation Oncology Anhui Provincial Cancer Hospital Hefei Anhui P. R. China

**Keywords:** organelle crosstalk, oxidative stress, radio‐immunotherapy, radiosensitivity, radiotherapy, reactive oxygen species, redox homeostasis, tumor immunity

## Abstract

Radiotherapy is a major modality for cancer treatment, but therapeutic responses vary substantially across tumor types and patients. Beyond direct DNA damage, radiation‐induced reactive oxygen species (ROS) are increasingly recognized as dynamic regulators of cellular stress responses. Their biological effects depend not only on the magnitude of ROS generation, but also on its duration, subcellular distribution, and whether antioxidant, repair, and organelle quality‐control systems can contain the resulting damage. Transient oxidative stress may activate adaptive responses, whereas persistent ROS can trigger mitochondrial ROS amplification, ER stress and Ca^2+^ dysregulation, lysosomal dysfunction, iron‐dependent lipid peroxidation, and irreversible cell injury. These processes are coordinated through an interconnected organelle network rather than occurring independently. ROS also exerts context‐dependent effects on antitumor immunity: localized oxidative stress can promote danger‐associated molecular patterns (DAMPs) release, cGAS–STING signaling, and immune activation, whereas prolonged oxidative stress may impair effector immune cells and favor immunosuppression. This review integrates these mechanisms within an organelle‐level redox framework and discusses emerging strategies for improving radiotherapy and radioimmunotherapy through selective modulation of redox homeostasis, with particular attention to tumor selectivity, safety, hypoxia, and clinical translation.

## Significance Statement

- Radiation‐induced ROS are not simply damaging by‐products but dynamic signals that determine whether irradiated cells adapt or progress toward irreversible injury. This review integrates mitochondrial ROS amplification, ER stress and Ca^2+^ signaling, lysosomal quality control, ferroptosis, and innate immune activation into a unified multi‐organelle redox framework. It further highlights the dual immunological effects of oxidative stress and discusses how tumor‐selective and temporally controlled redox modulation may improve radiotherapy and radioimmunotherapy while limiting normal‐tissue toxicity.

## 1 Introduction

Ionizing radiation damages tumor cells through both direct ionization of DNA and other macromolecules and indirect reactions initiated by water radiolysis [1, 2]. The latter rapidly generates highly reactive species such as •OH. Radiation also perturbs mitochondrial electron transport, increasing electron leakage and providing a secondary source of reactive oxygen species (ROS) after the initial irradiation event [3]. Thus, radiosensitivity is influenced not only by the extent of DNA damage and repair, but also by whether oxidative stress is rapidly resolved or persists after irradiation [4].

This distinction is important because ROS can support either recovery or irreversible injury [5]. Transient oxidative stress activates antioxidant defenses, DNA repair, autophagy, and mitophagy, whereas sustained ROS gradually overwhelms these protective systems [6, 7]. There is therefore no single ROS concentration that defines the transition between adaptation and cell death [8]. What matters is whether ROS production and damage accumulation remain within the capacity of antioxidant buffering, molecular repair, and organelle quality control [9, 10]. In colorectal cancer, for instance, SIRT3‐dependent PINK1/Parkin mitophagy removes dysfunctional, ROS‐producing mitochondria and limits prolonged mitochondrial oxidative stress, thereby contributing to radioresistance [11].

Much of this regulation occurs through crosstalk among mitochondria, the endoplasmic reticulum (ER), and lysosomes [12, 13]. Mitochondrial electron‐transport dysfunction sustains mitochondrial reactive oxygen species (mtROS) production, while prolonged ER‐to‐mitochondrial Ca^2+^ transfer further increases mitochondrial metabolic stress [14]. If ER stress is not resolved, the unfolded protein response (UPR) can shift toward PERK–ATF4–CHOP‐associated terminal signaling [15, 16]. Lysosomes have a dual role: autophagy and mitophagy remove damaged cellular components, whereas NCOA4‐mediated ferritinophagy increases the labile Fe^2+^ pool and promotes lipid peroxidation and ferroptosis [17, 18]. This latter pathway has been demonstrated in glioblastoma, where combined carbon‐ion and photon irradiation induced cytosolic dsDNA accumulation and cGAS–STING activation, followed by NCOA4–FTH1‐dependent ferritinophagy, Fe^2+^ accumulation, and enhanced lipid peroxidation [19]. Evidence for a direct role of Golgi remodeling or extracellular‐vesicles (EVs) biogenesis in radiosensitivity is less established; at present, ER‐to‐Golgi STING trafficking provides the clearest mechanistic link between Golgi function and radiation‐induced stress signaling [20].

Oxidative stress also influences the immune response to radiotherapy [21]. Mitochondrial DNA and nuclear DNA fragments that accumulate in the cytosol can activate cGAS–STING, induce type I interferons, and promote dendritic‐cell activation and CD8^+^ T‐cell priming [22]. The same process can become unfavorable when oxidative stress is prolonged or widespread, because T cells are themselves vulnerable to mitochondrial dysfunction and lipid peroxidation [23]. The view of radiation‐induced ROS has therefore expanded from short‐lived mediators of molecular damage to regulators of organelle stress, cell fate, and antitumor immunity (Figure 1). In this review, we examine how mitochondrial ROS amplification, ER stress and Ca^2+^ signaling, lysosomal quality control, ferroptosis, and innate immune signaling shape radiation response. We also discuss approaches that increase tumor oxidative stress, weaken antioxidant and stress‐adaptive pathways, interfere with organelle quality control, or enhance radioimmunotherapy, with particular attention to tumor selectivity, normal‐tissue toxicity, hypoxia, and patient selection.

**FIGURE 1:**
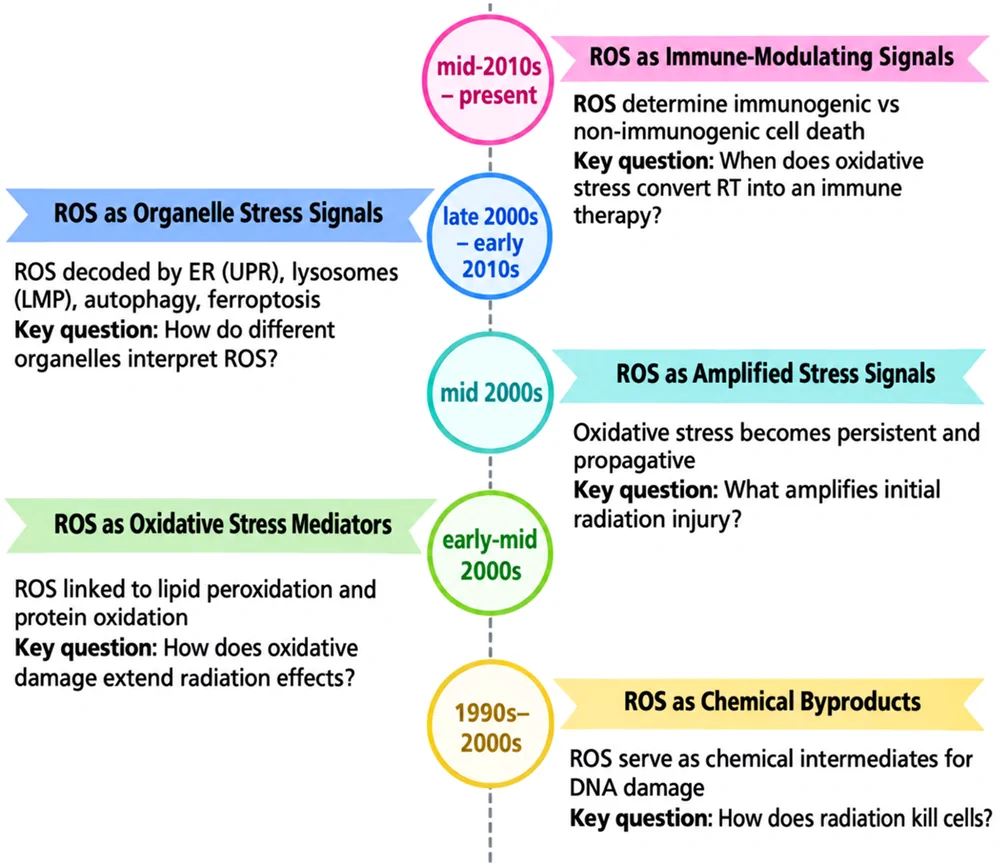
Evolution of the understanding of radiotherapy‐induced ROS. The timeline illustrates the conceptual progression from ROS as short‐lived chemical intermediates of radiation damage, to mediators of oxidative stress, self‐amplifying stress signals, organelle‐specific regulators, and finally immune‐modulating signals. This evolution reflects a broader understanding of ROS as dynamic determinants of radiosensitivity, cell‐fate decisions, and antitumor immunity. Created in BioRender. Ju, G. (2026), https://BioRender.com/qutow4g.

## 2 Radiotherapy and ROS

Ionizing radiation induces cellular damage through both direct and indirect mechanisms. Direct ionization can damage DNA and other biomolecules, whereas indirect effects are largely mediated by the radiolysis of intracellular water, which rapidly generates ROS [24]. Meanwhile, radiation can also interfere with mitochondrial electron transfer processes and increase electron leakage [14]. Together, water radiolysis and radiation‐induced mitochondrial dysfunction contribute to the rapid increase in intracellular ROS after irradiation. The temporal evolution of these oxidative events, from the initial radiation‐induced ROS burst to mitochondrial ROS amplification and persistent oxidative stress, is summarized in Figure 2.

**FIGURE 2:**
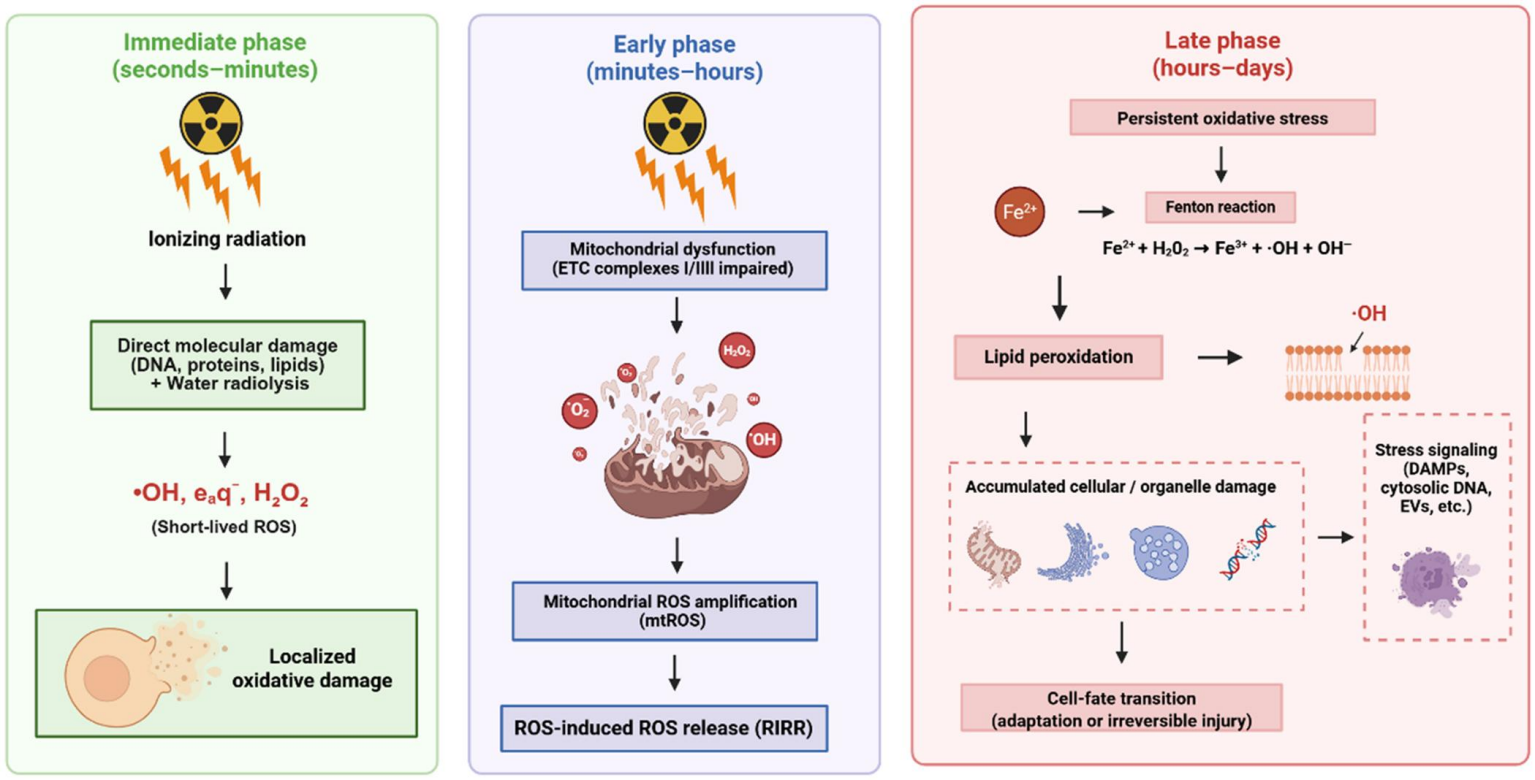
Temporal evolution of radiation‐induced oxidative stress. Immediate radiation injury arises from direct molecular damage and water radiolysis, followed by mitochondrial ROS amplification and ROS‐induced ROS release. Persistent oxidative stress subsequently promotes Fe^2+^‐dependent Fenton chemistry, lipid peroxidation, and cumulative cellular/organelle damage, shifting the response from adaptation toward irreversible injury. Created in BioRender. Ju, G. (2026), https://BioRender.com/d8x6vge.

Radiotherapy produces hydroxyl radicals (•OH), superoxide anions (O_2_^‐^), and hydrogen peroxide (H_2_O_2_) [4, 25]. These ROS differ in their chemical properties and in how they behave inside cells. •OH is highly reactive but extremely short‐lived, and therefore reacts primarily with nearby DNA, proteins, and membrane lipids, producing local and instantaneous molecular damage [26]. In contrast, $O_{2}^{•-}$ and particularly H_2_O_2_ are relatively more stable and can participate in subsequent redox reactions, allowing the initial oxidative insult to develop into a more sustained intracellular stress response [3].

The changes in ROS after radiotherapy have clear temporal characteristics, as shown in Figure 2. In the initial stage of irradiation, ROS mainly arise from water radiolysis, with •OH representing an important short‐lived species. Because of its limited diffusion distance, the resulting oxidative damage is largely confined to the vicinity of the radiation track [2]. As radiation‐induced injury develops, mitochondrial electron transport chain (ETC) function, particularly at complexes I and III, becomes impaired. Disrupted electron flow increases electron leakage, allowing electrons to prematurely reduce molecular oxygen and generate $O_{2}^{•-}$ [14]. Mitochondrial superoxide can subsequently be converted into relatively stable H_2_O_2_, while mtROS further oxidize ETC components and mitochondrial membranes, causing additional electron leakage. This establishes a ROS‐induced ROS release cycle in which mitochondria progressively become a sustained source of oxidative stress [27].

In addition, changes in intracellular iron homeostasis can also affect the sustained presence of ROS. During radiation therapy, an increase in the labile Fe^2+^ pool can promote the Fenton reaction, in which H_2_O_2_ is converted into highly reactive •OH [28]. This process regenerates short‐lived radicals and promotes additional local oxidative damage and lipid peroxidation [29]. Together, mitochondrial ROS amplification and iron‐dependent Fenton chemistry can therefore convert the initial, transient radiation‐induced ROS burst into a more persistent and self‐amplifying oxidative stress state [28]. Thus, the critical determinant of the radiation response may not simply be the magnitude of the initial ROS burst, but whether this oxidative signal is rapidly terminated or develops into sustained intracellular ROS amplification.

Cells counteract radiation‐induced oxidative stress through both antioxidant and damage‐control systems. Superoxide dismutases convert $O_{2}^{•-}$ into H_2_O_2_, whereas catalase, the glutathione system, and related peroxidases remove H_2_O_2_ and lipid hydroperoxides [30]. In parallel, DNA repair, autophagy, and mitochondrial quality‐control pathways remove damaged macromolecules and organelles [31]. When these systems keep pace with ROS production and damage accumulation, redox homeostasis can be restored and cells may recover after irradiation [5].

On the contrary, when the production rate of ROS continues to exceed the cell's clearance capacity, the balance gradually shifts from protective negative‐feedback regulation toward self‐amplifying positive‐feedback loops [5, 9]. Persistent mitochondrial damage increases mtROS production [27]; ER stress can disturb Ca^2+^ homeostasis and further increase mitochondrial oxidative burden [32]; and lysosomal iron release can enhance Fenton chemistry and lipid peroxidation [29]. These processes simultaneously increase ROS production and weaken the systems responsible for damage clearance.

Based on this phenomenon, a ROS threshold model has been proposed to describe the differences in cellular responses at different ROS levels [8]. Importantly, this threshold should not be interpreted as a fixed or universal ROS concentration [3]. Rather, it represents the dynamic point at which ROS production and damage accumulation exceed the combined antioxidant, repair, and organelle quality‐control capacity of the cell [33]. Low or moderate levels of ROS are often associated with stress adaptation, while sustained or excessive ROS are more likely to push cells into an irreversible state of damage [34]. Below this threshold, adaptive mechanisms dominate and favor restoration of homeostasis; once the threshold is crossed, mutually reinforcing organelle dysfunction makes redox recovery increasingly difficult and drives the transition toward irreversible cellular injury and cell death.

## 3 ROS and Organelle Responses

On the basis of the ROS dynamics described above, Figure 3 illustrates how transient radiation‐induced oxidative stress can develop into a sustained intracellular response. Early ROS are initially localized, but disruption of the mitochondrial electron transport chain increases electron leakage and mtROS production, establishing ROS‐induced ROS release [35]. Mitochondria therefore act as an early amplification hub, whereas the ER and autophagy–lysosome system determine whether this stress can still be buffered through Ca^2+^ regulation, proteostasis, and damaged‐organelle clearance [36]. When these adaptive mechanisms are sufficient, redox homeostasis can be restored; when they are overwhelmed, mitochondrial dysfunction, ER Ca^2+^ dysregulation, defective mitophagy, lysosomal injury, and iron‐dependent lipid peroxidation reinforce one another [37]. The Golgi/vesicular system may act further downstream by transmitting stress‐associated signals. Thus, radiation‐induced ROS is coordinated through a functional organelle hierarchy rather than through independent organelle responses. This hierarchical organization of radiation‐induced oxidative stress and the transition from adaptive buffering to persistent organelle dysfunction are summarized in Figure 3.

**FIGURE 3:**
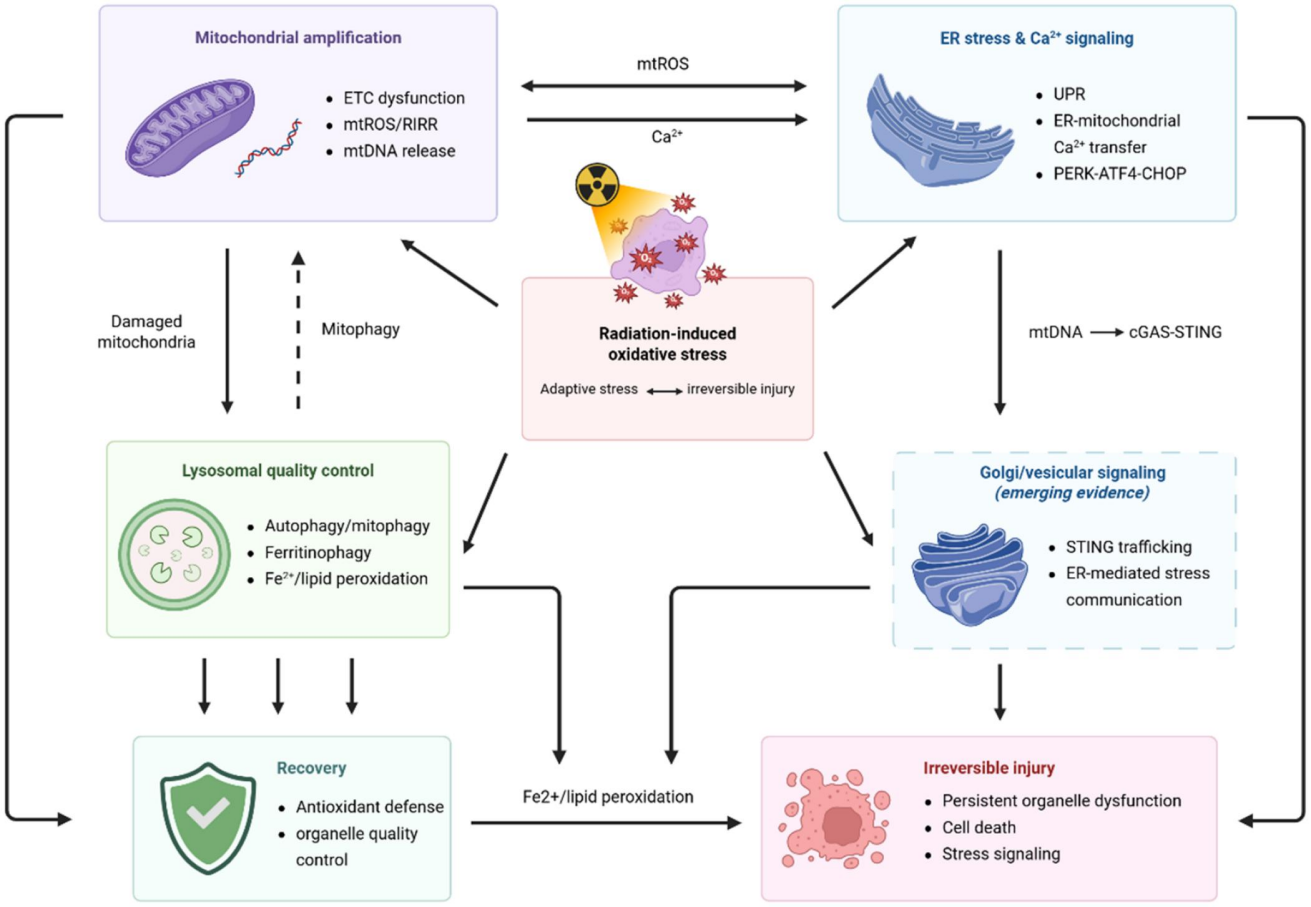
Hierarchical organelle responses to radiation‐induced oxidative stress. Mitochondria act as an early ROS‐amplification hub, whereas the ER and lysosomal system determine whether oxidative stress is buffered or progresses toward irreversible injury. Effective antioxidant and organelle quality‐control responses favor recovery, while persistent mitochondrial dysfunction, ER stress, and iron‐dependent lipid peroxidation promote cell death. Golgi/vesicular signaling is included as an emerging component of this stress network. Created in BioRender. Ju, G. (2026) https://BioRender.com/t55e9fe.

### 3.1 Mitochondrial ROS Amplification and Stress Adaptation

Mitochondria are an important source of intracellular oxidative stress after radiotherapy. Radiation‐induced disruption of the mitochondrial electron transport chain increases electron leakage and promotes mtROS production, which can further impair mitochondrial function and amplify oxidative stress [35, 38].

However, increased mtROS does not necessarily lead directly to radiosensitization. When mitochondrial injury remains limited, damaged mitochondria can be selectively removed through mitophagy, thereby reducing the number of ROS‐producing organelles [39]. Moderate or transient mtROS can also activate antioxidant and mitochondrial quality‐control programs that facilitate recovery after irradiation. For example, SIRT3‐dependent activation of PINK1/Parkin‐mediated mitophagy has been shown to promote radioresistance in colorectal cancer cells by enhancing the clearance of dysfunctional mitochondria and facilitating recovery from radiation‐induced damage [11]. Similarly, NEAT1v1 promoted PINK1/Parkin‐mediated mitophagy and reduced radiation‐induced mitochondrial oxidative stress in hepatocellular carcinoma cells. These findings support the concept that mitochondrial oxidative stress can remain within an adaptive range when mitochondrial quality‐control capacity is sufficient to match the rate of damage [11, 39].

When mitochondrial damage persists, however, ETC dysfunction and mtROS production reinforce each other [35]. Once damage accumulates faster than dysfunctional mitochondria can be repaired or removed, mitochondrial membrane potential declines and outer mitochondrial membrane permeability increases [38]. Cytochrome c and Smac/Diablo can subsequently be released into the cytosol, initiating caspase‐dependent apoptotic signaling [40]. Persistent lipid peroxidation and metabolic dysfunction may also favor other regulated cell‐death programs, depending on antioxidant buffering capacity, iron availability, and the metabolic state of tumor cells [19, 41]. Thus, the transition from adaptation to irreversible injury is determined not simply by mtROS abundance, but by whether mitochondrial damage exceeds the capacity of antioxidant and quality‐control systems [11, 39].

Mitochondrial oxidative stress can also influence immune signaling after radiotherapy. Persistent mitochondrial membrane damage and disturbed mitochondrial dynamics can promote mtDNA release or mislocalization into the cytosol [22]. Cytosolic mtDNA can be recognized by cGAS, leading to STING activation and downstream TBK1–IRF3/type I interferon signaling [22]. This process is closely linked to mitochondrial quality control: efficient mitophagy can remove damaged mitochondria before excessive mtDNA accumulates, whereas failure of mitophagic clearance allows both mtROS and cytosolic mtDNA to increase [39]. Mitochondrial dysfunction can therefore shift from a reversible metabolic disturbance toward irreversible injury or an inflammatory and immunogenic signal, depending on the balance between damage generation and mitochondrial quality control.

### 3.2 Redox Imbalance and Stress Signaling in the Endoplasmic Reticulum

Research has shown that the redox homeostasis of the endoplasmic reticulum is highly sensitive to radiation‐induced stress, and its imbalance can trigger a wide range of signaling cascades [42]. The endoplasmic reticulum provides the intracellular environment required for protein maturation and lipid production. This environment depends on a tightly regulated redox balance. Following radiotherapy, cellular ROS levels rise and this balance is disturbed, as reported previously. Within the endoplasmic reticulum, oxidation of cysteine residues becomes more frequent. This interferes with normal disulfide‐bond formation and protein folding, leading to the accumulation of misfolded and unfolded proteins within the ER lumen [43]. This accumulation is sensed by the cell and leads to activation of the UPR. In the early stages of stress, UPR mainly manifests as adaptive regulation. PERK activation reduces global protein synthesis and rapidly lowers the amount of newly synthesized proteins entering the ER, whereas IRE1α‐dependent signaling enhances protein‐folding and degradation programs [44, 45]. UPR‐associated autophagy further contributes to the removal of damaged proteins and organelles [46]. In cells with strong antioxidant capacity or high levels of basal stress, this state can be maintained for a certain period of time. At this stage, the UPR therefore acts primarily as a buffering mechanism that allows tumor cells to restore proteostasis and survive radiation‐induced stress [42, 44].

When protein‐folding demand continues to exceed the adaptive capacity of the UPR, the response shifts from proteostatic recovery toward terminal ER stress. Continued protein oxidation causes the folding burden to exceed the adaptive capacity of the UPR. The UPR signal strength further increases, and its functional orientation changes. The PERK–ATF4–CHOP‐related pathway is continuously activated, and the balance of survival and death signals in cells changes accordingly [47, 48].

Persistent ER stress also disrupts Ca^2+^ homeostasis. Increased Ca^2+^ transfer from the ER to mitochondria initially can support mitochondrial metabolism, but sustained mitochondrial Ca^2+^ loading increases respiratory‐chain burden, electron leakage, and mtROS generation [49]. Consequently, ROS aggravates ER stress while ER‐derived Ca^2+^ further amplifies mitochondrial oxidative stress, forming an ER–mitochondrial positive‐feedback loop that promotes the transition from adaptive stress to irreversible damage [50].

At the same time, endoplasmic reticulum stress can also affect signaling features on the cell surface, such as the translocation of calreticulin to the plasma membrane. Surface‐exposed calreticulin functions as an “eat‐me” signal that facilitates recognition of stressed tumor cells by antigen‐presenting cells. ATP and HMGB1 may subsequently be released as cellular injury progresses, further contributing to immune recognition [51]. Thus, the ER acts as an important stress‐transition node: an early adaptive UPR can promote recovery, whereas failure of proteostasis and Ca^2+^ regulation reinforces mitochondrial dysfunction and promotes irreversible and potentially immunogenic cellular injury.

### 3.3 Lysosomes and Autophagy

As reported previously, lysosomes are more sensitive to oxidative stress, and their membrane structure is rich in unsaturated lipids, making them more prone to lipid peroxidation. After radiation therapy, an increase in ROS levels can weaken the stability of lysosomal membranes and gradually increase membrane permeability [52]. As membrane integrity is compromised, the contents of lysosomes begin to enter the cytoplasm, including various proteases. Limited lysosomal membrane damage may remain reversible, whereas extensive lysosomal membrane permeabilization (LMP) allows cathepsins and other hydrolases to accumulate in the cytosol and participate in cell‐death signaling [52].

Even under mild stress, lysosomes can still maintain their degradation function. Through autophagy, damaged mitochondria and oxidized biomolecules are cleared, thereby alleviating the oxidative burden within the cell [53].

Mitophagy is particularly important in this process because successful lysosomal degradation of damaged mitochondria directly removes a major source of sustained mtROS [39]. Thus, when autophagic flux and lysosomal degradative capacity are sufficient to match the amount of radiation‐induced damage, autophagy primarily functions as a cytoprotective response [42].

When oxidative stress persists, the regulatory ability of lysosomes gradually becomes limited, and their functional orientation changes accordingly. Persistent lysosomal injury can impair autophagic flux: autophagosomes may continue to form, but their cargo cannot be efficiently degraded [54]. Damaged mitochondria therefore accumulate and continue to produce mtROS. Consequently, increased autophagy markers after irradiation do not necessarily indicate effective protective autophagy; the critical determinant is whether complete autophagic flux and lysosomal degradation remain intact.

Iron metabolism plays a crucial role in this process. Lysosomes are important sites for ferritin degradation, and radiation exposure can accelerate this process, thereby increasing intracellular free iron levels [55]. The released Fe^2+^ reacts with H_2_O_2_ through Fenton chemistry to generate additional •OH, which accelerates phospholipid peroxidation. Under normal conditions, the glutathione–GPX4 system reduces lipid hydroperoxides and prevents their accumulation. When iron‐dependent lipid peroxide production exceeds this antioxidant capacity, however, membrane phospholipid peroxides accumulate and drive ferroptosis [19].

Therefore, the effect of autophagy after irradiation depends not simply on its activation level, but on whether autophagic flux is completed and on the specific cargo being degraded. Removal of damaged mitochondria is generally protective, whereas selective degradation of ferritin or antioxidant proteins can increase iron‐dependent lipid peroxidation and promote ferroptosis [39]. Thus, the autophagy–lysosome system represents another critical stress threshold. Intact autophagic flux removes ROS‐producing damaged organelles and favors survival, whereas lysosomal failure simultaneously prevents damage clearance and enhances iron‐dependent ROS production, converting a protective degradation pathway into an amplifier of irreversible oxidative injury.

### 3.4 Golgi Apparatus and Redox‐Controlled Stress Propagation

Compared with mitochondria, the ER, and lysosomes, the role of the Golgi apparatus in radiation‐induced oxidative stress is less well established. Radiation therapy–induced oxidative stress has been reported to affect the structural stability of the Golgi apparatus, and an increase in ROS levels can weaken the integrity of the Golgi apparatus membrane and interfere with the maintenance of its bandlike structure. As the structure is damaged, the Golgi apparatus is more prone to fragmentation, which also affects protein processing, sorting, and vesicular trafficking.

Cellular oxidative stress has also been associated with changes in extracellular‐vesicle production and cargo composition [56]. Stress‐associated vesicles may contain oxidized lipids, mitochondrial components, proteins, and nucleic acids [57]. However, current evidence does not establish a simple causal sequence in which radiation‐induced ROS directly induces Golgi fragmentation and subsequently increases extracellular‐vesicle biogenesis. These observations should therefore be interpreted as potentially related processes rather than as a fully established radiotherapy pathway.

After the release of vesicles, oxidative stress‐related information can diffuse between cells, causing disturbances that were originally confined to the interior of the cell to spread outward. Once released, stress‐associated extracellular vesicles may transmit oxidized lipids, mitochondrial components, proteins, and nucleic acids to neighboring cells, thereby extending oxidative stress beyond the irradiated cell [56, 57, 58]. Oxidative stress has also been associated with alterations in Rab‐dependent vesicular trafficking and cytoskeletal transport, which may influence the release and distribution of stress‐associated cargo. However, whether these changes directly contribute to tumor radiosensitivity remains unclear [59].

These findings suggest that vesicular trafficking may provide a route through which stress signals initially generated by mitochondrial, ER, or lysosomal dysfunction are transmitted to neighboring tumor, stromal, or immune cells [60]. The Golgi/vesicular system may therefore be positioned downstream of the more established organelle stress pathways as a potential mediator of intercellular stress propagation. Nevertheless, its direct causal contribution to tumor radiosensitivity remains incompletely defined and requires further experimental validation.

## 4 Organelle Crosstalk Network

Radiation‐induced ROS is not confined to one organelle. Mitochondrial damage increases electron leakage from the respiratory chain and maintains mtROS production after the initial oxidative burst [35]. The ER is closely involved because sustained Ca^2+^ transfer to mitochondria further increases mitochondrial stress and ROS generation [49]. Damaged mitochondria can be removed by PINK1/Parkin‐dependent mitophagy and lysosomal degradation, which helps limit further ROS accumulation. This protective effect is lost when mitochondrial damage exceeds the capacity of mitophagic clearance [11]. Lysosomes can then contribute to oxidative injury through NCOA4‐mediated ferritinophagy, which increases the labile Fe^2+^ pool and promotes Fenton chemistry, lipid peroxidation, and ferroptosis [19]. The Golgi appears to participate mainly at a later stage. ER‐to‐Golgi STING trafficking is relatively well established, whereas the roles of Golgi remodeling and extracellular‐vesicle signaling in radiosensitivity remain less clear. Figure 4 summarizes these interactions and shows how organelle responses can either restrict oxidative injury or allow it to become self‐sustaining.

**FIGURE 4:**
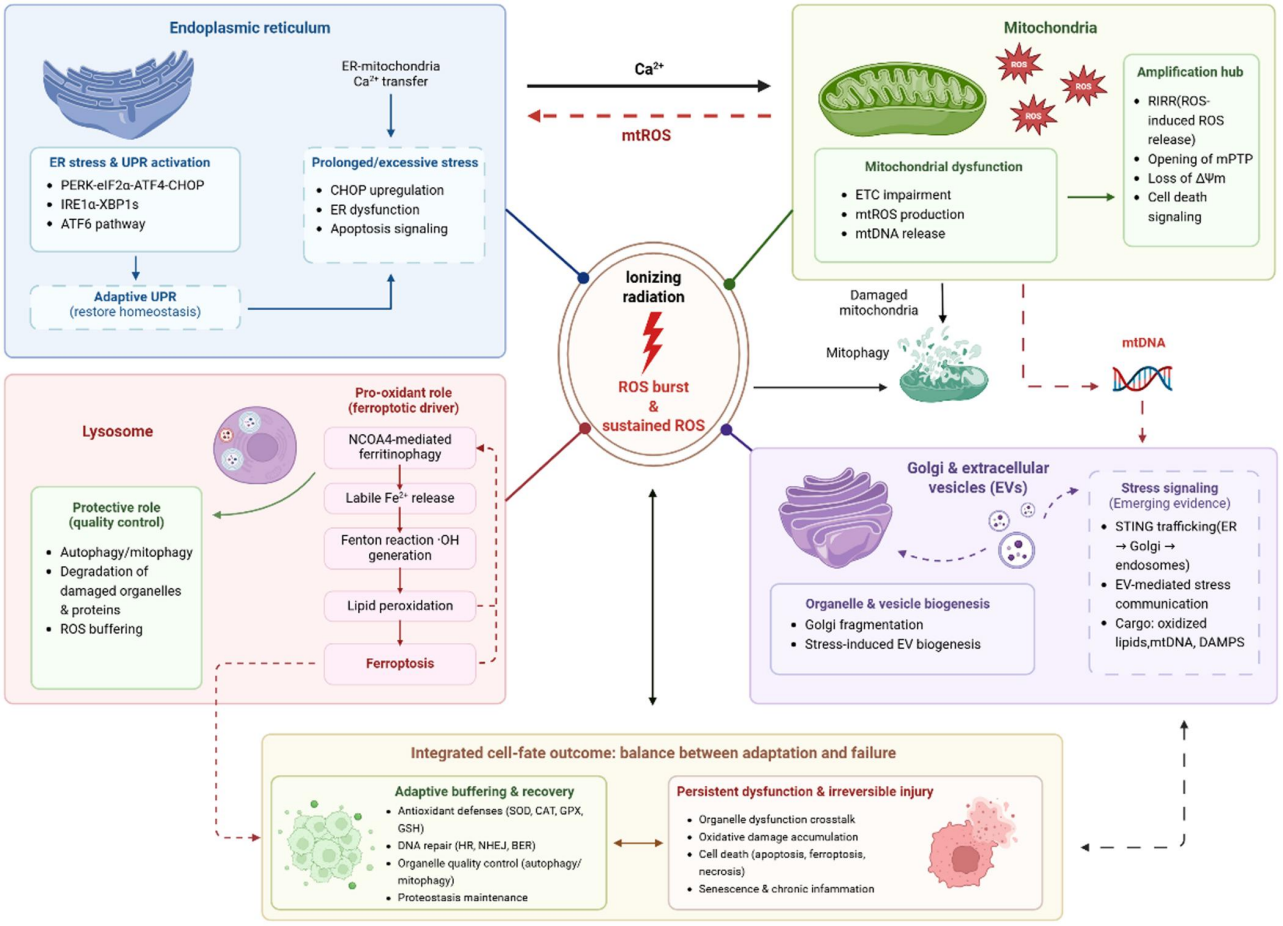
Multi‐organelle crosstalk in radiation‐induced redox stress. Radiation‐induced ROS are amplified by mitochondrial dysfunction and further propagated through ER–mitochondrial Ca^2+^ exchange. Lysosomes can either limit oxidative injury through autophagy/mitophagy or enhance it through NCOA4‐mediated ferritinophagy, Fe^2+^ release, lipid peroxidation, and ferroptosis. Mitochondrial DNA release and ER‐to‐Golgi STING trafficking connect organelle damage with downstream stress signaling, whereas EV‐mediated communication remains less established. The balance between antioxidant/repair capacity and persistent organelle dysfunction ultimately determines adaptation vs. irreversible cell injury. Created in BioRender. Ju, G. (2026) https://BioRender.com/bjk9kqb.

### 4.1 Mitochondria–ER Contact Sites as a Redox‐Sensitive Control Hub

Mitochondria‐associated membranes (MAMs) are regions of close contact between mitochondria and the ER and provide an important platform for Ca^2+^ exchange. At these sites, ER‐derived Ca^2+^ can be transferred through the IP3R–GRP75–VDAC1 axis and subsequently enter mitochondria through the mitochondrial calcium uniporter (MCU) [61]. Under moderate stress, controlled mitochondrial Ca^2+^ uptake supports oxidative phosphorylation and ATP production.

When oxidative stress persists, however, Ca^2+^ influx can exceed mitochondrial buffering capacity, increasing respiratory‐chain burden, electron leakage, and mtROS production. mtROS further aggravates ER stress and Ca^2+^ dysregulation, establishing a ROS–Ca^2+^ positive‐feedback loop [49]. Progressive mitochondrial dysfunction then increases membrane permeability and promotes mtDNA release into the cytosol [22].

Cytosolic mtDNA activates cGAS and promotes cGAMP‐dependent STING signaling [22]. STING subsequently traffics from the ER to the Golgi, where TBK1–IRF3 signaling is activated and type I interferon production is induced [62]. Thus, ER–mitochondrial Ca^2+^ coupling and ER–Golgi STING trafficking provide a direct mechanistic bridge from oxidative organelle stress to innate immune activation.

### 4.2 ER–Lysosome Coordinates Proteostasis, Autophagy, and Cell Death

During moderate oxidative stress, the unfolded protein response (UPR) reduces the protein‐folding burden, while the autophagy–lysosome system removes damaged proteins and organelles [42]. Mitophagy links mitochondrial stress directly to lysosomal clearance by eliminating dysfunctional mitochondria before persistent mtROS accumulates [39]. When ER stress and mitochondrial damage continue to increase, however, lysosomal degradation may no longer keep pace with the damage burden. Incomplete autophagic flux then allows damaged mitochondria to accumulate, further increasing mtROS and reinforcing ER stress [42].

Lysosomal iron metabolism provides an additional route for oxidative amplification. NCOA4‐mediated ferritinophagy increases the labile Fe^2+^ pool and promotes Fenton‐driven lipid peroxidation [55]. When lipid‐peroxide generation exceeds glutathione–GPX4‐dependent detoxification, ferroptosis becomes increasingly favored [55]. A recent glioblastoma study provides a representative example of this transition: combined carbon‐ion and photon irradiation induced cytosolic dsDNA accumulation and cGAS–STING activation, which promoted NCOA4–FTH1‐dependent ferritinophagy, increased Fe^2+^ and lipid peroxidation, and ultimately enhanced ferroptosis [19]. This finding illustrates how radiation‐induced stress signaling can converge on lysosomal iron metabolism and convert a damage response into a self‐amplifying ferroptotic state.

Thus, lysosomes occupy a dual position in the redox network: they suppress oxidative stress by clearing damaged mitochondria, but can also amplify oxidative injury through iron release and lipid peroxidation [39, 55]. The outcome therefore depends on the balance between degradative capacity, mitochondrial damage, and iron‐dependent oxidative stress.

### 4.3 Golgi Apparatus and Vesicular Pathways in Redox‐Dependent Stress Communication

Compared with mitochondria, the ER, and lysosomes, the role of the Golgi apparatus in radiation‐induced redox regulation remains less well established. A more clearly defined connection is provided by STING trafficking. Following cGAS activation, STING translocates from the ER to the Golgi, where downstream TBK1–IRF3 signaling is organized. This places the Golgi at an interface between intracellular organelle stress and innate immune activation [62].

Extracellular‐vesicle‐mediated signaling may provide an additional route for stress communication. Vesicles released from irradiated or oxidatively stressed tumor cells can contain oxidized lipids, mitochondrial components, proteins, and nucleic acids, thereby transmitting stress‐associated cargo to neighboring cells [56, 57]. However, direct evidence that radiation‐induced Golgi remodeling drives extracellular‐vesicle production or independently determines radiosensitivity remains limited [59]. The Golgi/vesicular pathway should therefore currently be regarded as an emerging component of the radiation‐induced redox network rather than a primary determinant of radiation response.

## 5 ROS and Immune Regulation

Radiation‐induced organelle stress can expose or release intracellular danger signals and thereby connect oxidative injury with antitumor immunity [22]. However, ROS is not uniformly immunostimulatory. Transient and tumor‐restricted oxidative stress can enhance immune recognition, whereas persistent or widespread ROS may impair effector immune cells and favor an immunosuppressive microenvironment [63, 64]. Thus, the immune consequence of ROS depends on its magnitude, duration, spatial distribution, and cellular targets.

### 5.1 Radiation‐Induced ROS Modulate Cell Death and Immunogenicity

The immunogenicity of radiation‐induced cell death is not determined solely by ROS level or by the type of cell death, but also by the nature and amount of danger‐associated molecular patterns (DAMPs) exposed or released. Radiation‐induced ER stress can promote calreticulin exposure on the cell surface, whereas ATP and HMGB1 released during subsequent cellular injury facilitate immune‐cell recruitment, phagocytosis, and antigen presentation [51].

Persistent ROS can also promote iron‐dependent lipid peroxidation and ferroptosis [19]. However, ferroptosis is not invariably immunogenic. Oxidized phospholipids and other damage‐associated molecules may enhance inflammatory signaling, whereas excessive lipid oxidation can damage surrounding immune cells or generate immunosuppressive signals [65]. Its immune consequence therefore depends on the magnitude, spatial distribution, and cellular targets of lipid peroxidation rather than on ferroptosis alone [65].

Together, mitochondrial damage, ER stress, lysosomal permeabilization, and lipid peroxidation generate distinct classes of DAMPs. Their combined effects, rather than a single form of cell death, determine whether radiation‐induced injury ultimately promotes productive antitumor immunity [51, 65].

### 5.2 ROS‐Mediated Activation of Innate Immune Signaling Pathways

Mitochondrial damage can release mtDNA into the cytosol, where it is recognized by cGAS and activates STING‐dependent signaling [66]. ROS therefore promotes cGAS–STING mainly by increasing mitochondrial and nuclear damage rather than by directly activating cGAS. Downstream TBK1–IRF3 activation and type I interferon production subsequently link organelle injury to dendritic‐cell activation and T‐cell priming [22, 67]. A representative example comes from nasopharyngeal carcinoma, where irradiation‐induced mtDNA release was shown to activate STING signaling and CD8^+^ T‐cell‐mediated antitumor immunity. Mechanistically, TRIM21 promoted VDAC2 degradation and restricted mtDNA release, whereas TRIM21 loss enhanced cytosolic mtDNA accumulation, STING–TBK1–IRF3 signaling, antigen presentation, and the antitumor response to irradiation [22]. This finding illustrates that the extent of mitochondrial damage and mtDNA escape can determine whether radiation‐induced mitochondrial stress remains intracellular or is converted into an innate immune signal.

ROS also regulates NF‐κB‐ and NRF2‐dependent transcriptional programs. NF‐κB can support inflammatory cytokine production, whereas NRF2‐mediated antioxidant adaptation limits oxidative injury and may favor tumor‐cell survival [68, 69]. Increased mtROS, ionic imbalance, and lysosomal damage can additionally facilitate NLRP3 inflammasome activation and IL‐1β/IL‐18 maturation [70].

These innate immune pathways are themselves context‐dependent. Controlled cGAS–STING or inflammasome activation may enhance antitumor immunity, whereas persistent inflammatory signaling can promote tissue injury, myeloid‐cell recruitment, and tumor‐supportive inflammation [71]. Thus, the immune consequence of radiation‐induced organelle stress depends not only on pathway activation itself, but also on the magnitude and duration of the upstream damage signal.

### 5.3 ROS‐Mediated Remodeling and Immunomodulation of the Tumor Microenvironment

After radiotherapy, ROS and stress signals can persist in the tumor microenvironment. Early after irradiation, DAMP release, type I interferon signaling, and inflammatory chemokines support dendritic‐cell activation, antigen presentation, and recruitment of CD8^+^ T cells [72].

The situation changes when oxidative stress persists. Sustained ROS and lipid peroxidation impair T‐cell mitochondrial function, proliferation, and cytotoxicity. CD36‐mediated uptake of oxidized lipids, for example, increases lipid peroxidation in tumor‐infiltrating CD8^+^ T cells and suppresses their effector activity; reducing lipid peroxidation or blocking ferroptosis restores antitumor function and improves the response to PD‐1 blockade [64]. Ferroptotic PMN‐MDSCs provide another example: these cells release oxidized lipids that inhibit T‐cell activity. Thus, lipid peroxidation in immune cells can shift an irradiated tumor from an immune‐stimulatory state toward immune suppression [65]. Persistent oxidative stress can also reduce dendritic‐cell function and support suppressive myeloid responses [73].

Extracellular vesicles provide an additional route for transmitting oxidized nucleic acids, lipids, and proteins between cells [74]. Radiation‐induced vascular changes can also alter immune‐cell entry into irradiated tissues [75, 76]. The effect of these processes depends on the cargo being transferred, the recipient cell, and how long the oxidative stress persists.

This balance is especially important during immune‐checkpoint blockade. DAMP release and cGAS–STING/type I interferon signaling after irradiation can create a short period in which PD‐1/PD‐L1 or CTLA‐4 blockade is more effective [72, 77]. If oxidative stress persists, however, T‐cell dysfunction and myeloid‐mediated suppression can reduce this benefit [64]. Radioimmunotherapy may therefore work best when oxidative stress is strong enough to expose tumor danger signals but remains sufficiently localized and transient to avoid damaging effector immune cells. Figure 5 summarizes this shift. Low or efficiently buffered ROS is associated with limited immune visibility; intermediate, tumor‐restricted oxidative stress favors DAMP release, cGAS–STING/type I interferon signaling, dendritic‐cell activation, and CD8^+^ T‐cell priming; persistent or widespread ROS increasingly damages immune cells and promotes suppressive myeloid responses.

**FIGURE 5:**
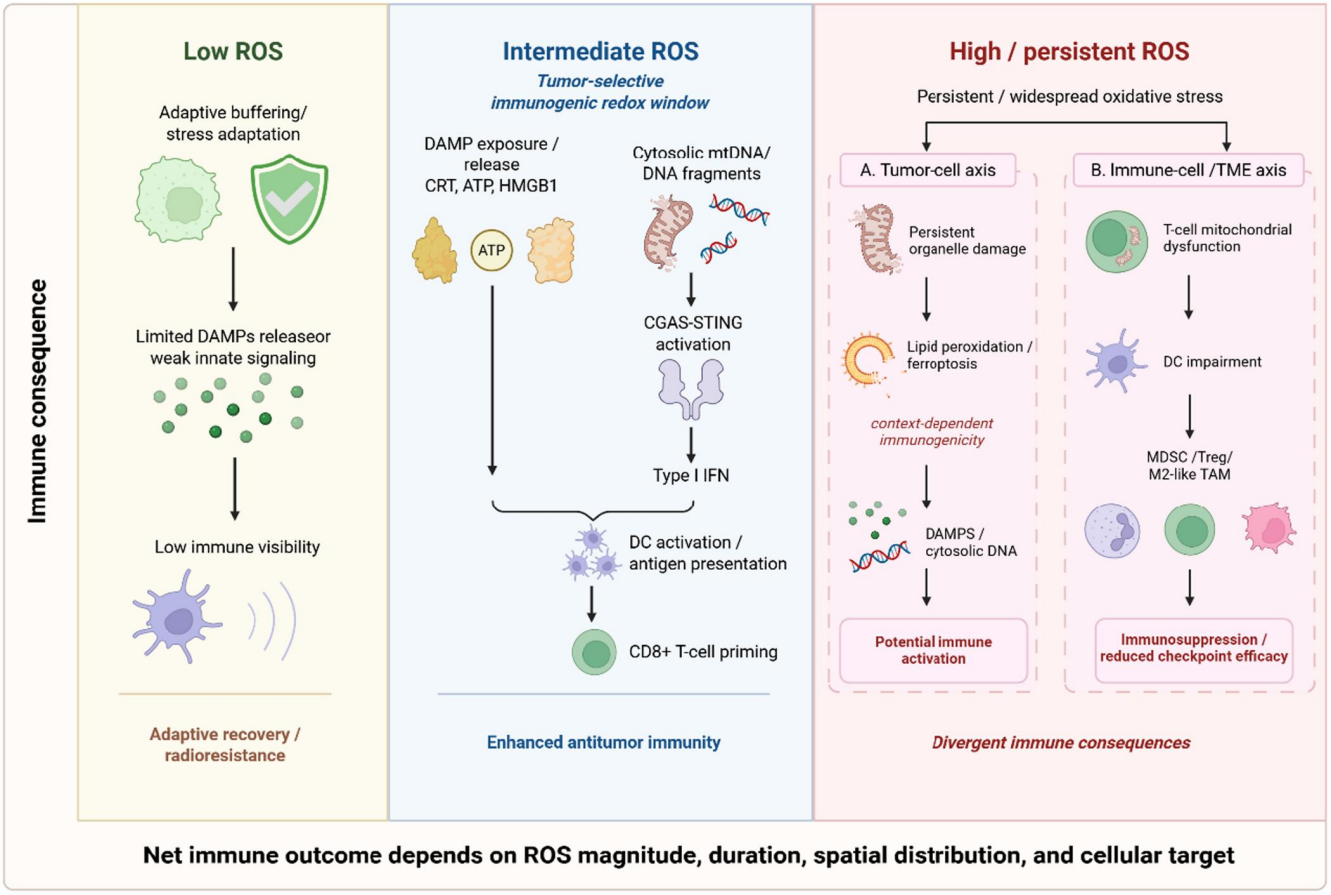
Context‐dependent immune consequences of radiation‐induced ROS. Low ROS favors adaptive recovery with limited immune visibility, whereas intermediate, tumor‐restricted oxidative stress promotes DAMP release, cytosolic DNA accumulation, cGAS–STING/type I IFN signaling, dendritic‐cell activation, and CD8^+^ T‐cell priming. High or persistent ROS produces divergent effects: tumor‐cell damage may enhance immunogenic signaling, while oxidative injury to immune cells can promote T‐cell dysfunction, dendritic‐cell impairment, and immunosuppressive myeloid responses. The final immune outcome depends on ROS magnitude, duration, spatial distribution, and cellular targets. Created in BioRender. Ju, G. (2026) https://BioRender.com/2plfnmc.

Representative recent studies linking organelle‐specific redox mechanisms to radiation response and translational relevance are summarized in Table 1.

**TABLE 1:** Representative recent studies linking redox stress to radiation response and translational relevance.

Mechanistic category	Representative study	ROS source	Key mechanism	Functional role in radiation response	Translational relevance
Mitophagy/radioresistance	Wei et al., 2023 [11]	Radiation‐induced mtROS	SIRT3 activates PINK1/Parkin‐dependent mitophagy and enhances damage repair	Adaptive; radioresistance	Patient association; potential radiosensitization target
Mitochondrial metabolism/hypoxia	Rondeau et al., 2024 [78]	Mitochondrial O_2_ consumption	MitoQ reduces mitochondrial oxygen consumption and improves tumor oxygenation	Radiosensitizing	Drug‐repurposing potential in hypoxic tumors
ER–mitochondrial Ca^2+^ stress	Lai et al., 2026 [50]	Ca^2+^‐driven ROS amplification	ER Ca^2+^ dysregulation sustains ROS and ER stress, promoting apoptosis	Damage‐amplifying; radiosensitizing	Recent pharmacological proof‐of‐concept
Ferroptosis/patient stratification	Noh et al., 2025 [79]	Lipid ROS	A ferroptosis‐resistant signature limits radiation response, whereas statins restore ferroptotic sensitivity	Radiosensitizing	Patient stratification and drug repurposing
Ferritinophagy/ferroptosis	Chen et al., 2025 [19]	Fe^2+^‐dependent lipid ROS	cGAS–STING promotes NCOA4–FTH1‐dependent ferritinophagy, increasing Fe^2+^ and lipid peroxidation	Damage‐amplifying; ferroptosis	Relevant to carbon‐ion/photon RT optimization
Nanocatalytic ROS amplification	Hu et al., 2024 [80]	Radiation and catalytic ROS	HfO_2_@MnO_2_@GOx increases ROS, depletes GSH, promotes ferroptosis, and activates cGAS–STING	Radiosensitizing; immunogenic	Multifunctional theranostic platform; preclinical
Catalytic redox modulation	Yang et al., 2025 [81]	Catalytic ROS and hypoxia	Mn‐based catalysis relieves hypoxia, amplifies ROS, and promotes ferroptosis	Radiosensitizing; immunogenic	Targeted radionuclide‐radioimmunotherapy potential
EV‐mediated tumor microenvironment (TME) modulation	Liang et al., 2024 [82]	Radiation‐induced stress signaling	Radiation enhances EV tumor targeting, while EV‐mediated TGF‐β blockade remodels the irradiated TME	Enhances RT response	Emerging EV‐based radioimmunotherapy strategy
Normal‐tissue mitochondrial protection	Jiao et al., 2024 [83]	Radiation‐induced mtROS	SGLT1 inhibition enhances mitophagy and preserves mitochondrial homeostasis	Radioprotection	Potential normal‐tissue protection strategy
Normal‐tissue ferroptosis	Li et al., 2026 [84]	Fe^2+^/lipid ROS	NCOA4‐mediated ferritinophagy increases labile iron and promotes lipid peroxidation and ferroptosis	Normal‐tissue injury	Defines safety limits of ferroptosis‐based radiosensitization

## 6 Therapeutic Implications and Future Directions

Radiotherapy produces substantial oxidative stress, but irradiated tumor cells can partly recover by activating antioxidant defenses and other stress‐adaptive pathways [11, 68]. This recovery limits the effect of radiotherapy alone and creates several points for therapeutic intervention, including further increasing tumor ROS, weakening antioxidant buffering, interfering with organelle quality control, and enhancing antitumor immune responses [85]. The aim is to make oxidative damage exceed the tolerance of tumor cells without causing comparable injury to normal tissues [33]. Figure 6 summarizes these intervention strategies, and Table 2 lists representative ROS‐targeting approaches together with their mechanisms, therapeutic effects, and translational status.

**FIGURE 6:**
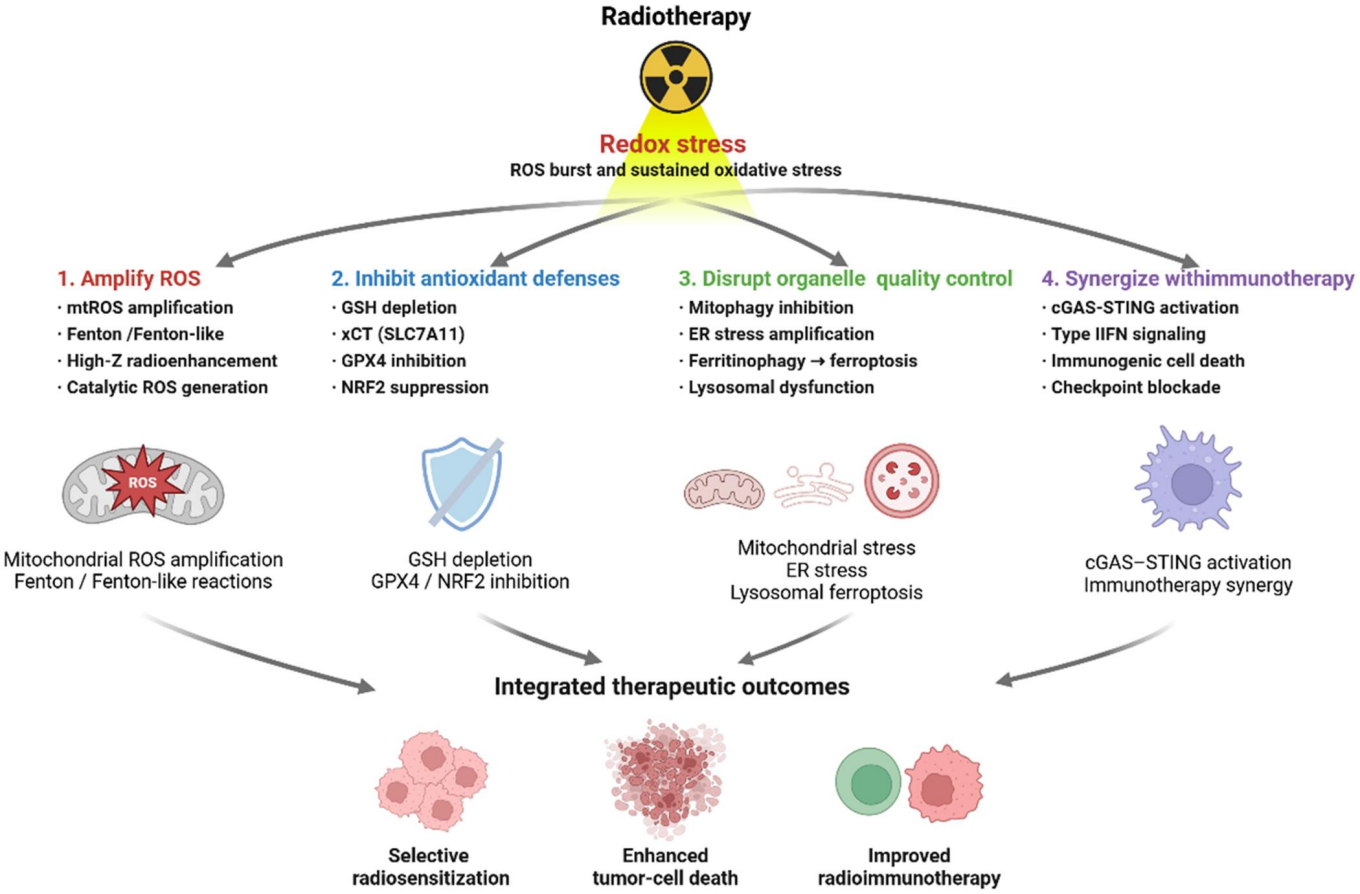
Strategies for targeting ROS dynamic changes to enhance radiation therapy efficacy. By regulating ROS production levels, antioxidant capacity, organelle stress response, and synergistic effects with immunotherapy, it affects the radiation‐induced cell death mode and anti‐tumor immune response. Created in BioRender. Ju, G. (2026) https://BioRender.com/v6kqy2j.

**TABLE 2:** ROS‐targeting strategies for enhancing radiotherapy and radioimmunotherapy.

Strategy category	Representative study	Redox target	Key mechanism	Therapeutic effect	Translational status/limitation
ROS amplification	Allen et al., 2019 [85]	H_2_O_2_/redox‐active iron	Enhances peroxide‐dependent oxidative stress	Radiosensitization	Phase I in GBM; clinically feasible
High‐Z radioenhancement	Bonvalot et al., 2019 [86]	Radiation‐induced radicals	HfO_2_ increases local energy deposition and radical generation	Enhanced RT response	Randomized Phase II–III evidence; intratumoral administration
High‐Z radioenhancement	Verry et al., 2021 [87]	Radiation‐induced radicals	Gd‐based nanoparticles enhance radiation energy deposition	Radiosensitization	Phase I clinical evidence; heterogeneous tumor uptake
Antioxidant inhibition	Skeie et al., 2026 [88]	xCT/GSH	Reduces cystine uptake and GSH‐dependent ROS buffering	Radiosensitization	Phase I in recurrent GBM; intratumoral GSH reduction demonstrated
Ferroptosis induction	Lei et al., 2020 [89]	Lipid‐peroxide detoxification	Weakens lipid‐peroxide clearance and promotes ferroptosis	Radiosensitization	Mainly preclinical; normal‐tissue and immune‐cell toxicity remain concerns
Organelle quality‐control targeting	Wei et al., 2023 [11]	Damaged mitochondria	Limits clearance of radiation‐damaged mitochondria and sustains mtROS	Overcomes adaptive radioresistance	Preclinical; strongly context‐dependent
Innate immune amplification	Cooper et al., 2025 [90]	cGAS–STING/type I IFN	Amplifies radiation‐induced STING‐dependent immune signaling	Enhanced radioimmunotherapy	Phase Ib with RT + pembrolizumab; early clinical efficacy

### 6.1 Enhancing ROS Generation and Amplification

One approach to radiosensitization is to increase radiation‐induced oxidative stress beyond the buffering capacity of tumor cells. This can be achieved by enhancing endogenous ROS production, introducing additional peroxide stress, or increasing local radical generation during and after irradiation. Importantly, the therapeutic goal is not to maximize ROS indiscriminately, but to preferentially amplify oxidative stress within irradiated tumors while limiting damage to surrounding normal tissues [85].

Mitochondria provide an important endogenous source of ROS amplification. Inhibition of respiratory‐chain complexes I or III increases electron leakage and mitochondrial ROS production, which can further impair mitochondrial function and prolong oxidative stress after irradiation [14, 35]. Pharmacologic ascorbate increases the oxidative burden through H_2_O_2_ generation; in the presence of redox‐active iron, H_2_O_2_ can further produce highly reactive radicals and reinforce radiation‐induced damage. Nanoparticle radioenhancers provide a more spatially confined means of enhancing radiation effects. NBTXR3, a hafnium oxide nanoparticle, increases secondary electron production during irradiation, thereby enhancing water radiolysis and local radical formation [86]. AGuIX (Activation and Guidance of Irradiation by X‐ray), a gadolinium‐based nanoparticle, enhances radiation‐induced ROS through a similar high‐atomic‐number effect [87].

ROS amplification can also continue after the initial radiation event. Metal‐based nanomaterials containing iron, copper, or manganese can amplify tumor oxidative stress through catalytic redox reactions, antioxidant depletion, or radiation‐enhanced energy deposition [80]. For example, pH‐sensitive Dy/Mn‐P nanoparticles markedly increased ROS generation during irradiation and promoted cytosolic DNA accumulation and STING activation, linking local ROS amplification with innate immune signaling [91]. Bimetallic Cu^2+^/Hf^4+^‐based nanostimulators further combined Hf‐mediated radioenhancement with GSH depletion, hypoxia relief, and Cu‐dependent cuproptosis, thereby reinforcing radiation‐induced oxidative damage and antitumor immunity [92]. Once oxidative damage extends to membrane lipids, Fe^2+^‐dependent radical reactions can further propagate lipid peroxidation. If lipid‐peroxide generation exceeds GPX4‐dependent detoxification, the process can progress toward ferroptosis [89, 93]. Together, these approaches increase either the magnitude or the duration of radiation‐induced oxidative damage, making redox recovery more difficult and increasing the likelihood that tumor cells exceed their oxidative tolerance.

### 6.2 Inhibiting Antioxidant Defenses and Stress Adaptation

Another way to increase radiosensitivity is to weaken the antioxidant systems that normally clear radiation‐induced ROS. Tumor cells depend mainly on the glutathione (GSH), thioredoxin (TXN), and peroxiredoxin (PRDX) systems to maintain redox balance. When these defenses are reduced, ROS persists after irradiation and oxidative damage accumulates more readily [68].

These pathways can be targeted at several levels. Buthionine sulfoximine (BSO) inhibits glutamate‐cysteine ligase and lowers intracellular GSH, reducing peroxide detoxification [94]. In glioblastoma, sulfasalazine‐mediated xCT inhibition reduced tumor GSH and was explored clinically as a means of increasing radiosensitivity [88]. Other targets act further downstream. SOD2 inhibition favors mitochondrial ROS accumulation, whereas GPX4 inhibition prevents phospholipid hydroperoxide detoxification and promotes ferroptosis [89]. NRF2 supports a broader antioxidant response after irradiation; suppression of the NRF2–SLC7A11 axis reduces GSH and increases ROS and lipid peroxidation [68, 95].

Recovery from irradiation also requires removal of damaged proteins and organelles. The unfolded protein response (UPR) helps restore proteostasis, while autophagy and mitophagy clear oxidized components and dysfunctional mitochondria. Blocking these pathways can prolong oxidative stress in tumors that rely on them for recovery [42]. Their effects are not uniform, however, because selective autophagy can also promote ferroptosis, for example through NCOA4‐mediated ferritin degradation [19, 55]. Thus, radiosensitization can be achieved not only by generating more ROS, but also by preventing tumor cells from detoxifying ROS and repairing the damage it causes.

### 6.3 Manipulating Organelle Signaling and Cell Fate

Mitochondrial and lysosomal quality control directly affects how long oxidative damage persists after irradiation. Damaged mitochondria are an important source of secondary ROS, while lysosomes help remove dysfunctional organelles and damaged cellular components.

Mitophagy is one route by which tumor cells reduce the mitochondrial ROS burden after irradiation. In nasopharyngeal carcinoma, irradiation increased LACTB2 and activated PINK1‐dependent mitophagy; blocking LACTB2 or PINK1 made the cells more radiosensitive [96]. In hepatocellular carcinoma, NEAT1v1 promoted PINK1/Parkin‐mediated mitophagy and reduced radiation‐induced mitochondrial oxidative stress [39]. SIRT3 has a similar role in colorectal cancer, where activation of PINK1/Parkin mitophagy supports recovery after irradiation [11]. In these tumors, removal of dysfunctional mitochondria limits continued mtROS production and favors survival after radiation exposure.

Lysosomal ferritin degradation has a different consequence. NCOA4 transports ferritin to lysosomes, where its degradation releases Fe^2+^ into the labile iron pool. The additional iron supports Fenton chemistry and lipid peroxidation [55]. This pathway was observed in glioblastoma treated with combined carbon‐ion and photon irradiation: cytosolic DNA accumulation activated cGAS–STING, followed by NCOA4–FTH1 ferritinophagy, Fe^2+^ accumulation, lipid peroxidation, and ferroptosis [19]. Mitophagy therefore removes a source of ROS, whereas ferritinophagy can provide the iron needed to amplify oxidative damage. Their contribution to radiosensitivity will differ according to which process is active in a particular tumor.

### 6.4 Synergizing Immunotherapy With ROS Modulation

Radiation‐induced oxidative damage can influence antitumor immunity by promoting DAMP release and cytosolic DNA accumulation, which can activate cGAS–STING signaling, induce type I interferons, and enhance dendritic‐cell activation and T‐cell priming [51, 72, 97]. This provides a mechanistic basis for combining radiotherapy with immune‐checkpoint blockade.

Clinical studies combining radiotherapy with immune‐checkpoint blockade provide indirect support for this therapeutic concept. In the PEMBRO‐RT trial, stereotactic body radiotherapy administered before pembrolizumab increased responses in nonirradiated lesions in metastatic non‐small‐cell lung cancer, with a more pronounced effect in tumors with low PD‐L1 expression [98]. Although this trial did not directly establish ROS or cGAS–STING signaling as the underlying mechanism, radiation‐induced oxidative and DNA damage may contribute to DAMP release and cytosolic DNA accumulation, thereby enhancing innate immune sensing before checkpoint inhibition. A more direct approach is to amplify this pathway pharmacologically. Dazostinag, an intravenously administered STING agonist, has been combined with hypofractionated radiotherapy and pembrolizumab [90], while the non‐nucleotide STING agonist diABZI enhanced radiation‐induced dendritic‐cell activation and PD‐1 blockade in preclinical models [99]. Nanomaterial‐based immune modulation provides another promising strategy to strengthen innate immune activation. For example, Mn^2+^/CpG oligodeoxynucleotide (ODN)‐codecorated black phosphorus nanosheets enabled pH‐responsive release of Mn^2+^ and CpG ODNs, simultaneously activating STING and Toll‐like receptor 9 (TLR9) signaling and thereby promoting macrophage reprogramming, dendritic‐cell activation, T‐cell infiltration, systemic antitumor immunity, and durable immune memory [100]. Together, these approaches illustrate how direct STING activation or complementary innate immune stimulation can strengthen downstream interferon signaling and antitumor immunity.

However, ROS does not invariably favor antitumor immunity. Persistent oxidative stress and lipid peroxidation can impair mitochondrial function and membrane homeostasis in activated T cells, reducing their proliferation and cytotoxic activity [64]. Such oxidative immune dysfunction may limit the benefit of radioimmunotherapy in some settings. Consistent with the broader clinical variability of this approach, adding the PD‐L1 inhibitor avelumab to chemoradiotherapy in the JAVELIN Head and Neck 100 trial failed to improve outcomes [101]. Thus, ROS modulation is most likely to enhance radioimmunotherapy when oxidative damage remains predominantly tumor‐restricted and is sufficient to trigger innate immune signaling without compromising effector immune cells.

## 7 Clinical Translation of Redox‐Based Radiosensitization

Clinical translation of redox‐based radiosensitization depends not only on whether a pathway can enhance radiation‐induced oxidative damage, but also on whether it can be modulated selectively, safely, and in the appropriate patient population. At present, drug repurposing, hypoxia modification, and nanoparticle radioenhancement are closest to clinical application, whereas direct targeting of GPX4, ferritinophagy, or mitophagy remains largely preclinical [11, 19, 85, 86, 87, 88, 89, 102].

### 7.1 Clinically Translatable Redox‐Modulating Strategies

Several redox‐modulating strategies have already entered clinical testing. Pharmacologic ascorbate has been combined with radiotherapy and temozolomide in newly diagnosed glioblastoma. By increasing H_2_O_2_‐dependent oxidative stress, pharmacologic ascorbate adds to the oxidative burden generated during irradiation [103]. A first‐in‐human phase I study showed that this combination was feasible and well tolerated, supporting further evaluation of pharmacological ROS amplification as a radiosensitizing strategy [85].

Targeting antioxidant dependence has also reached patients. Sulfasalazine inhibits the xCT cystine transporter, limiting glutathione synthesis and thereby weakening the ability of glioblastoma cells to buffer radiation‐induced ROS. In a 2026 phase I study in recurrent glioblastoma, short‐course sulfasalazine before stereotactic radiosurgery reduced intratumoral glutathione and was well tolerated [88]. These findings provide clinical proof‐of‐mechanism that tumor antioxidant capacity can be pharmacologically lowered before irradiation, although larger randomized studies are still required to establish therapeutic benefit.

Nanoparticle radioenhancers represent another clinically advanced strategy because they can increase radiation effects within the treated lesion without requiring systemic ROS elevation. NBTXR3 is an intratumorally administered hafnium oxide nanoparticle radioenhancer [86]. In the randomized phase II–III Act.In.Sarc trial in locally advanced soft‐tissue sarcoma, NBTXR3 combined with radiotherapy increased pathological complete response compared with radiotherapy alone [86]. AGuIX (Activation and Guidance of Irradiation by X‐ray), a gadolinium‐based theranostic nanoparticle, provides a systemically administered alternative and has also progressed into clinical evaluation with radiotherapy [87].

These examples illustrate why radiotherapy alone may be insufficient in tumors with strong redox adaptation. Pharmacologic or nanoparticle‐based interventions can either increase radiation‐induced oxidative stress or weaken the ability of tumor cells to recover from it. By contrast, direct GPX4 inhibition, NCOA4‐mediated ferritinophagy modulation, and PINK1/Parkin‐dependent mitophagy targeting remain primarily experimental because tumor selectivity and normal‐tissue tolerance have not yet been adequately established [11, 19, 89].

### 7.2 Safety and Tumor Selectivity

Whether ferroptosis‐inducing strategies can be safely combined with radiotherapy remains a major translational question. Radiation itself promotes ROS generation, iron‐dependent lipid oxidation, and ferroptotic signaling. Inhibiting xCT or GPX4 can further increase lipid‐peroxide accumulation in tumor cells, but the same antioxidant systems are also required to protect irradiated normal tissues [89].

This problem is directly illustrated by radiation‐induced intestinal injury. Ionizing radiation increased nuclear receptor coactivator 4 (NCOA4)‐mediated ferritinophagy in intestinal epithelial cells, leading to ferritin degradation, intracellular iron accumulation, mitochondrial ROS production, and ferroptosis. NCOA4 knockdown, iron chelation, or suppression of ferritinophagy reduced this injury [55]. Radiation‐induced NCOA4‐dependent ferritinophagy has also been implicated in endothelial injury, further indicating that systemic enhancement of ferroptosis could narrow the normal‐tissue therapeutic window [104, 105].

Tumor selectivity is therefore central to the safe use of redox‐based radiosensitizers. Nanoparticles offer one solution by restricting radiation enhancement spatially. NBTXR3 is delivered directly into the tumor and becomes active during irradiation [86], whereas systemically administered platforms such as AGuIX rely on preferential tumor accumulation [87]. Radiation‐responsive carriers and tumor‐specific catalytic nanomaterials provide additional preclinical approaches in which ROS amplification or ferroptosis induction occurs preferentially within the irradiated lesion [80]. The main challenge is to achieve sufficient intratumoral distribution without increasing oxidative injury in surrounding normal tissues.

Temporal selectivity is equally important. Antioxidant depletion is most useful when it overlaps with the radiation‐induced ROS burst, whereas prolonged GPX4 inhibition or iron‐dependent lipid oxidation may continue after irradiation and increase damage to normal tissues and infiltrating immune cells [64]. Safe ferroptosis‐based radiosensitization will therefore require both spatial and temporal control rather than maximal systemic ferroptosis induction.

### 7.3 Hypoxia, Patient Selection, and Remaining Challenges

Hypoxia is one of the clearest examples of how the tumor redox state determines radiotherapy response. Oxygen stabilizes radiation‐induced radical damage; when oxygen is limited, a greater proportion of this damage remains chemically reversible and tumor cells become less radiosensitive [106, 107].

Nimorazole was developed to overcome this problem by acting as an electron‐affinic radiosensitizer in hypoxic cells. In the randomized DAHANCA 5 trial, nimorazole improved locoregional control when added to radiotherapy [102]. Subsequent analysis using a 15‐gene hypoxia classifier showed that the benefit was concentrated in more hypoxic tumors, whereas less hypoxic tumors derived little additional benefit [108]. However, the phase III NIMRAD trial, which prospectively evaluated nimorazole with intensity‐modulated radiotherapy in head and neck cancer, failed to improve locoregional control or survival, including in the molecularly defined hypoxic subgroup [109]. The contrasting results indicate that the presence of hypoxia alone is insufficient; hypoxia must also be a dominant and therapeutically reversible cause of radioresistance in the selected population.

The ARCON strategy, which combines accelerated radiotherapy with carbogen breathing and nicotinamide, approaches hypoxia from the opposite direction by increasing oxygen availability and reducing acute perfusion‐related hypoxia [110]. In a randomized trial in laryngeal cancer, 5‐year regional control improved from 86% to 93%, with the clearest benefit observed in hypoxic tumors. This supports the concept that correcting tumor oxygenation can improve radiation response when oxygen limitation is biologically relevant [111].

Mitochondrial metabolism provides another way to modify hypoxia. Atovaquone inhibits mitochondrial respiration and reduces oxygen consumption, leaving more oxygen available within the tumor for radiation‐induced radical fixation [112]. A clinical window study in non‐small‐cell lung cancer showed a substantial reduction in tumor hypoxic volume in most treated patients. Metformin has been investigated through a similar mechanism. In patients specifically selected for fluoroazomycin arabinoside positron‐emission tomography (FAZA‐PET)‐positive hypoxic cervical tumors, 1 week of metformin reduced fractional hypoxic volume from 44.4% to 34.2% [113]. In contrast, adding metformin to chemoradiotherapy in the unselected NRG‐LU001 stage III non‐small‐cell lung cancer population did not improve progression‐free or overall survival [114]. These findings emphasize that modifying a redox or metabolic pathway does not necessarily improve clinical outcome unless that pathway is a major driver of resistance in the selected tumor.

Redox states differ between patients and across regions within the same tumor because oxygenation, antioxidant capacity, metabolic activity, and drug exposure are not uniform [115]. They also change during fractionated radiotherapy, as surviving tumor cells can activate NRF2‐dependent antioxidant responses, glutathione synthesis, autophagy, and mitophagy [11, 42, 68, 116]. These spatial and temporal changes make treatment timing important. Antioxidant depletion should be established before or during irradiation [88], nanoparticle radioenhancers must be present in the tumor at the time of radiation exposure [86], whereas metabolic modifiers require sufficient pretreatment to alter mitochondrial oxygen consumption and tumor oxygenation [112].

Overall, clinical translation of ROS‐based radiosensitization is unlikely to rely on indiscriminate ROS elevation. A more realistic strategy is therefore to identify the dominant redox constraint in each tumor—such as hypoxia, antioxidant dependence, lipid‐peroxide detoxification, or mitochondrial oxygen consumption—and select patients in whom that pathway is a major driver of radioresistance. The corresponding intervention should then be delivered within an acceptable normal‐tissue and immune‐cell therapeutic window.

## 8 Conclusion

Radiotherapy‐induced cellular responses extend beyond direct DNA damage and are strongly influenced by ROS‐mediated redox stress. Rather than acting only as damaging by‐products, ROS function as dynamic regulators of mitochondrial dysfunction, ER stress, lysosomal quality control, programmed cell death, and immune signaling [3, 34]. The biological consequence of ROS depends not simply on its abundance, but on its duration, subcellular distribution, and whether antioxidant, repair, and organelle quality‐control systems can contain the resulting damage.

These organelle responses are closely interconnected. Mitochondria can amplify oxidative stress through sustained mtROS production, ER–mitochondrial Ca^2+^ signaling can further propagate this stress, and lysosomal pathways can either limit damage through organelle clearance or enhance oxidative injury through ferritinophagy, iron release, and lipid peroxidation [19, 49]. When these adaptive mechanisms remain sufficient, cellular homeostasis may be restored; when damage exceeds their combined capacity, oxidative stress becomes self‐amplifying and favors irreversible injury, ferroptosis, apoptosis, and other forms of programmed cell death.

ROS also has context‐dependent effects on antitumor immunity. Transient and tumor‐restricted oxidative stress can promote DAMP release, cGAS–STING signaling, and immune activation, whereas persistent or widespread oxidative stress may impair effector immune cells and favor an immunosuppressive microenvironment [64, 72]. Therefore, the therapeutic goal should not be to maximize ROS indiscriminately, but to selectively push tumor cells beyond their adaptive redox capacity while preserving normal tissues and antitumor immune function.

Future clinical translation will depend on identifying the dominant redox vulnerability in individual tumors, such as hypoxia, antioxidant dependence, mitochondrial metabolism, or lipid‐peroxide detoxification, and matching it with spatially and temporally controlled interventions. This organelle‐level redox framework may provide a more integrated basis for understanding radiosensitivity and for developing more selective and effective radiotherapy and radioimmunotherapy strategies.

## Author Contributions

Guangyu Ju and Zimeng Wang contributed equally to this work. Guangyu Ju, Zimeng Wang, and Junchao Qian conceived the topic and developed the overall review framework. Jingjing Xiao, Qinxue Wu, Zhiying Yang, and Yuqing Miao performed the literature search, extracted relevant evidence, and supported conceptual refinement. Guangyu Ju drafted the initial manuscript. Zimeng Wang, Jingjing Xiao, Qinxue Wu, Zhiying Yang, and Yuqing Miao contributed to content organization and figure conceptualization. Junchao Qian and Shuanghu Yuan critically reviewed, edited, and supervised the manuscript development. All authors read and approved the final version of the manuscript.

## Conflicts of Interest

The authors declare no conflicts of interest.

## Data Availability

The authors have nothing to report.
